# Infliximab Induces Clonal Expansion of γδ-T Cells in Crohn's Disease: A Predictor of Lymphoma Risk?

**DOI:** 10.1371/journal.pone.0017890

**Published:** 2011-03-31

**Authors:** Jens Kelsen, Anders Dige, Heinrich Schwindt, Francesco D'Amore, Finn S. Pedersen, Jørgen Agnholt, Lisbet A. Christensen, Jens F. Dahlerup, Christian L. Hvas

**Affiliations:** 1 Gastro-Immuno Research Laboratory (GIRL), Department of Medicine V, Aarhus University Hospital, Aarhus, Denmark; 2 Institute of Molecular Biology, Aarhus University, Aarhus, Denmark; 3 Department of Hematology, Aarhus University Hospital, Aarhus, Denmark; Sheba Medical Center, Israel

## Abstract

**Background:**

Concominant with the widespread use of combined immunotherapy in the management of Crohn's disease (CD), the incidence of hepato-splenic gamma-delta (γδ)-T cell lymphoma has increased sharply in CD patients. Malignant transformation of lymphocytes is believed to be a multistep process resulting in the selection of malignant γδ-T cell clones. We hypothesised that repeated infusion of anti-TNF-α agents may induce clonal selection and that concurrent treatment with immunomodulators further predisposes patients to γδ-T cell expansion.

**Methodology/Principal Findings:**

We investigated dynamic changes in the γδ-T cells of patient with CD following treatment with infliximab (Remicade®; *n* = 20) or adalimumab (Humira®; *n* = 26) using flow cytometry. In patients with a high γδ-T cell level, the γδ-T cells were assessed for clonality. Of these 46 CD patients, 35 had a γδ-T cells level (mean 1.6%) comparable to healthy individuals (mean 2.2%), and 11 CD patients (24%) exhibited an increased level of γδ-T cells (5–15%). In the 18 patients also receiving thiopurines or methotrexate, the average baseline γδ-T cell level was 4.4%. In three male CD patients with a high baseline value, the γδ-T cell population increased dramatically following infliximab therapy. A fourth male patient also on infliximab monotherapy presented with 20% γδ-T cells, which increased to 25% shortly after treatment and was 36% between infusions. Clonality studies revealed an oligoclonal γδ-T cell pattern with dominant γδ-T cell clones. In support of our clinical findings, in vitro experiments showed a dose-dependent proliferative effect of anti-TNF-α agents on γδ-T cells.

**Conclusion/Significance:**

CD patients treated with immunomodulators had constitutively high levels of γδ-T cells. Infliximab exacerbated clonal γδ-T cell expansion in vivo and induced γδ-T cell proliferation in vitro. Overall, young, male CD patients with high baseline γδ-T cell levels may be at an increased risk of developing malignant γδ-T cell lymphomas following treatment with anti-TNF-α agents.

## Introduction

T cells that express the γδ subunits of the T cell receptor link innate and adaptive immunity and have been implicated in the pathogenesis of autoimmune diseases, particularly Crohn's disease (CD) [1]. The frequency of γδ-T cells in the peripheral blood of healthy individuals ranges from 2–5%. However, higher γδ-T cell frequencies have been found in CD patients [2], and these increased levels have been reported to mirror disease activity, with higher levels in patients with active disease[3]. Hepatosplenic T cell lymphoma (HSTCL) is a rare and distinct peripheral T cell lymphoma that is nearly always γδ-T cell in origin[4]. HSTCL has been observed in patients receiving immunosuppressive treatment and in a disproportionately high number of young male CD patients [5]–[7].

To date, approximately 200 cases of HSTCL have been reported worldwide. Interestingly, of these cases, 28 cases were reported in patients with inflammatory bowel disease (IBD). With the exception of one case, the occurrence of anti-TNF-α treatment–associated HSTCL has only been reported in IBD patients [5], [8]. Of these 28 cases, 22 patients had received infliximab in combination with a thiopurine analogue (azathioprine or 6-mercaptopurine) [9], 3 cases were associated with the use of infliximab followed by adalimumab. However, HSTCL was also reported to occur in patients receiving azathioprine monotherapy [10], [11].

In a large study conducted by CESAME, where only 9% of the patients received anti-TNF-α therapy, evidence for a possible causal role of thiopurines in lymphomagenesis was reported; however, no cases of HTSCL were reported [12]. HSTCL incidence is very low, and clinical trials may not provide a follow-up period that is sufficiently long enough to detect overt lymphoma development in the studied cohorts. Thus, a causal link between thiopurine treatment and the possible increased risk of combining thiopurine treatment with anti-TNF-α agents has been difficult to establish[13]. Furthermore, it is unknown if there are differences in the risk associated with the various anti-TNF-α agents. As a result of the observed HSTCL cases, a stepdown to monotherapy has been advocated in young IBD patients [14]. However, the lymphoma risk apparently persists, as even a single exposure to infliximab appears to predispose patients to lymphoma development years later [15].

The malignant transformation of lymphocyte subsets in IBD patients is believed to be a multistep process resulting in the selection of γδ-T cell clones with a survival advantage [16]. In this context, we hypothesised that repeated treatment with anti-TNF-α antibodies in a standard maintenance regimen may contribute to this process and that concomitant thiopurine treatment may further promote γδ-T cell expansion. Therefore, we examined circulating γδ-T cells in patients with active CD before and after treatment with the anti-TNF-α antibodies infliximab (Remicade®) and adalimumab (Humira®). We confirmed the hypothesis raised by previous epidemiological studies that infliximab has a profound proliferative effect on γδ-T cells both in vivo and in vitro and that infliximab treatment results in the clonal expansion of γδ-T cells in specific CD patients. We hypothesise that markedly elevated γδ-T cell levels may identify CD patients prone to develop lymphoproliferative disease during anti-TNF-α therapy.

## Materials and Methods

### Patients and Healthy Volunteers

We examined 46 patients with active CD, diagnosed based on internationally accepted clinical, histopathological, and biochemical criteria [17]. The patient characteristics are listed in Tables S1 and 1, and clinical disease activity was estimated using the Harvey-Bradshaw Index (HBI) [18]. Systemic inflammation was evaluated using C-reactive protein levels; faecal calprotectin [19] was used to estimate mucosal inflammation. Treatment decisions were based on the combined evaluation of clinical, biochemical, and faecal disease markers. In total, twenty patients were treated with infliximab (Remicade®, Centocor; 5 mg/kg intravenously at days 0, 14, and 42), and 26 patients were treated with adalimumab (Humira®, Abbott). The adalimumab-treated patients received a subcutaneous injection of 160 mg at week 0 and an injection of 80 mg at week 2. This was then followed by 40 mg maintenance injections every 2 weeks. Blood samples were obtained immediately before the administration of the anti-TNF-α agent and at days 1, 7, and 42 after the first treatment. One patient, number 47, was included post-hoc for further analyses and verification of our results. Sixteen healthy volunteers were recruited from the hospital staff to serve as controls. All participants provided written informed consent, and the study was approved by The Central Denmark Region Committee on Biomedical Research Ethics (j.no. 20040150).

### Whole-blood Flow Cytometry

For flow cytometric staining, 100 µl of venous blood was incubated for 20 minutes at room temperature with the optimised amounts of the following fluorescent-conjugated antibodies: anti-αβ-TCR-FITC (clone WT-31), anti-Vδ2-PE (clone B6), anti-CD3-PerCP (clone SK7), anti-CD8-PE-Cy7 (clone RPA-T8), anti-γδ-TCR-APC (clone B1), and anti-CD4-APC-Cy7 (clone RPA-T4). All antibodies were from BD Biosciences (San Diego, CA). After 10 minutes, the red blood cells were lysed using 2 ml Pharm Lyse Buffer (BD Biosciences), and the samples were centrifuged for 5 minutes at 200×*g* at 20°C. The washed cells were resuspended in 200 µl phosphate-buffered saline (PBS) with 2% pooled human AB serum and 1% formaldehyde. Six-colour flow cytometry was performed within 4 hours on a FACSCanto flow cytometer (BD Biosciences). For each sample, 30,000 events in the forward/side scatter live lymphocyte gate were recorded. All γδ-T cell frequencies are out of total CD3+ T cells. The data were analysed using FACSDiva 5.1 Software (BD Biosciences).

### Proliferation Assay

Peripheral blood mononuclear cells (PBMCs) were labelled with carboxyfluorescein succinimidyl ester (CFSE). The cells (1.5×10^6^ cells/ml) were cultured in RPMI 1640 supplemented with 10% human AB serum, penicillin/streptomycin, and rIL-2 (200 IU/ml). Cells were cultured in the absence or presence of infliximab (0.1 or 1.0 µg/ml), adalimumab (0.1 or 1.0 µg/ml) or etanercept (1.0 µg/ml). Ustekinumab (1.0 µg/ml), an antibody against IL-12/23(p40), was used as a control. Recombinant human TNF-α (10 ng/ml) (Genzyme, Cambridge, MA) was added to selected wells. Proliferation was measured on day 5 using flow cytometry, as previously described [20].

### Separation of γδ-T cells

PBMCs were isolated using Ficoll-Hypaque (GE Healthcare Bio-Sciences, Uppsala, Sweden) centrifugation, and γδ-T cells were purified using the TCRγ/δ T Cell Isolation Kit (Miltenyi Biotec, Bergisch Gladbach, Germany). Cell separation was performed on an AutoMACS Cell Separator, as recommended by the manufacturer. For all steps of the cell separation, we used PBS supplemented with 2 mM EDTA and 0.5% bovine serum albumin (BSA) (Sigma-Aldrich, Denmark). The purity of the γδ-T cells ranged between 90–95%.

### Preparation of Genomic DNA and Total RNA

For fragment analysis, genomic DNA was extracted from 2 ml of EDTA-treated whole blood according to the manufacturer's instructions (NucleoSpin Blood L, Macherey-Nagel, Germany). DNA was dissolved in 5 mM Tris/HCl, pH 8.5. The quality of DNA was assessed by PCR amplification of three fragments (195 bp, 450 bp, and 650 bp) of the p53 gene. Combined extraction of mRNA and genomic DNA from enriched γδ-T cell fractions was performed according to the manufacturer's instructions (AllPrep DNA/RNA Mini Kit, Qiagen, Germany). The quality of the genomic DNA was verified using PCR, as described above, while mRNA quality was assessed using gel electrophoresis.

### Multiplex PCR Assay

Identification of clonal populations with a specific T cell receptor delta (*TCRD*) rearrangement was performed according using the BIOMED-2 protocol [21], with slight modifications. PCR analysis of *TCRD* rearrangements was performed in a single tube with the *TCRD* primerset consisting of six Vδ and one Dδ2 (forward) primers or four Jδ and one Dδ3 primers (reverse) (Sigma Aldrich, St. Louis, MO, USA). Fluorescent labelling of the different Jδ and Dδ primers was done using HEX and 6FAM, respectively. The identification of clonal populations was performed by fragment analysis using a 3130xl genetic analyser and the Peak Scanner 1.0 Software (Applied Biosystems, Foster City, CA, USA). A clonal population was defined by the presence of a single peak or a predominant population. The fragment size was interpreted in accordance with the BIOMED-2 protocol. For all analyses, a second, confirmatory determination was performed.

### DNA Heteroduplex Analysis

To verify the fragment analysis results, PCR products were denatured at 95°C for 5 minutes and then re-annealed at 4°C for 1 hour. Heteroduplex products were separated using 6% non-denaturating polyacrylamide electrophoresis in 0.5× TBE-buffer, stained with 0.5 µg/ml ethidium bromide, and visualised using a UV-transilluminator.

### Statistical analysis

Both parametric and non-parametric statistical tests were used. Unpaired bivariate comparisons of continuous variables were carried out using the Student's *t*-test. Dichotomous variables were compared using the χ^2^ test. The mean and 95% confidence intervals (CI) are reported for continuous data. All correlations were evaluated using Spearman rho. A *p* value less than 0.05 was considered statistically significant. All statistical analyses were performed using SPSS 11.0 software.

## Results

### γδ-T cell Characteristics of CD Patients

**Figure 1:**
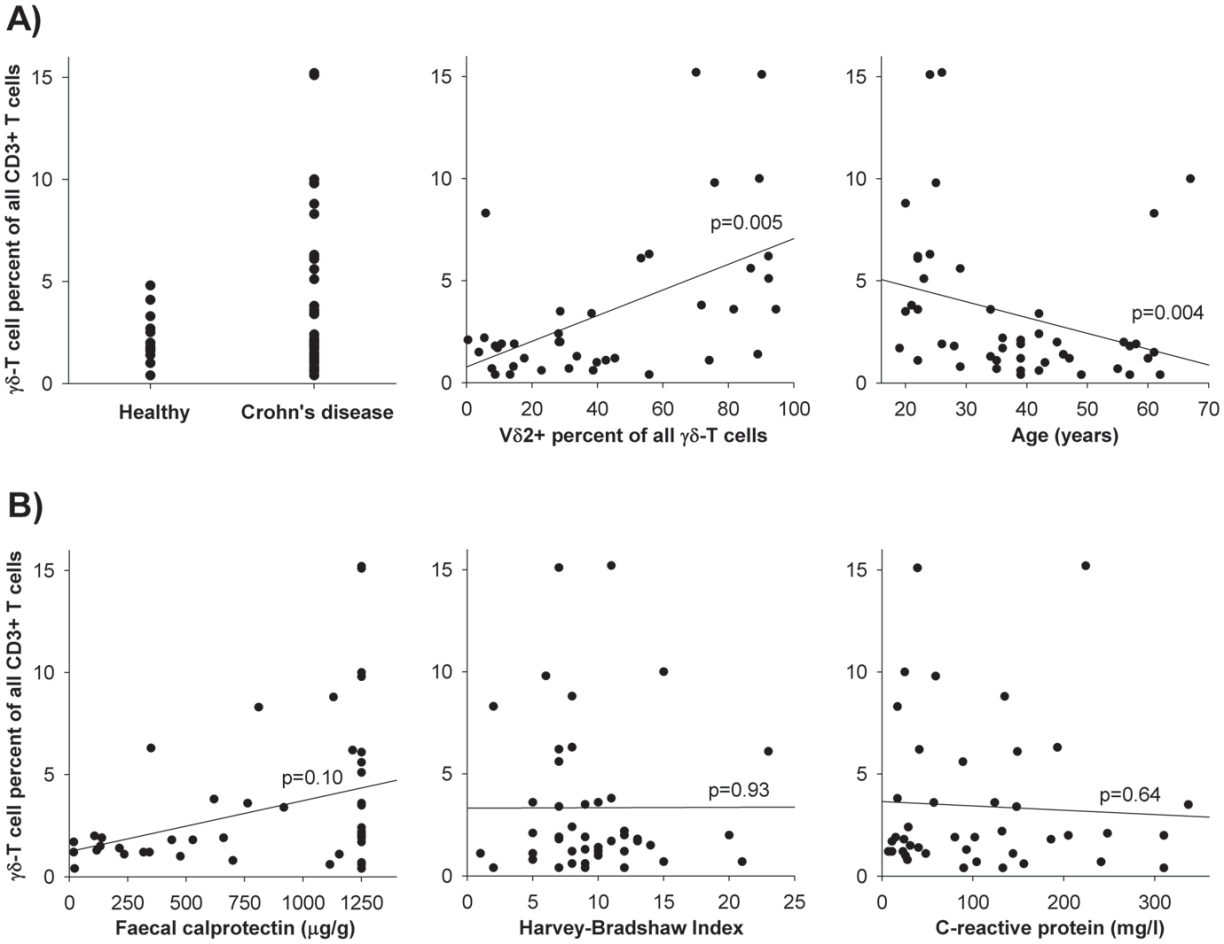
Baseline γδ-T cell characteristics in 46 CD patients. A) Of the 46 CD patients, 35 (76%) had a γδ-T cell level comparable to healthy volunteers, while 11 (24%) had a level ranging from 5 to 15% (left). The γδ-T cell level correlated with the expression of the Vδ2 subunit (middle), and the γδ-T cell levels correlated negatively with age (right). B) There were no statistically significant associations between γδ-T cell levels and markers of mucosal inflammation (faecal calprotectin), clinical disease activity (Harvey-Bradshaw Index), or systemic inflammation (C-reactive protein).

We recruited 46 CD patients with an even distribution of gender and age (Tables S1 and 1). At the time of analysis, 20 patients were being treated with infliximab and 26 with adalimumab. In the latter group, 11 patients (42%) had previously received infliximab. Of the 46 CD patients, 35 (76%) had a γδ-T cell level comparable to the level found in healthy volunteers. CD patients had a mean frequency of 1.6% γδ-T cells of total CD3+ T cells, with values ranging from 1.3–2.0%. Similarly, healthy volunteers had a mean γδ-T cell frequency of 2.2% with values ranging from 1.7–2.8%. While no healthy volunteer had a γδ-T cell level above 5%, 11 CD patients (24%) exhibited a high baseline percentage of γδ-T cells, with frequencies ranging from 5% to 15% Of these 11 patients, all were non-smokers (*p* = 0.008, χ^2^ test), and all had colonic inflammation (*p* = 0.14, χ^2^ test). Of the 11 patients, 8 were males (*p* = 0.12, χ^2^ test), and 7 were currently treated with azathioprine, 6-mercaptopurine, or methotrexate (*p* = 0.06, χ^2^ test). The γδ-T cell levels were negatively correlated with age (*p* = 0.004, Spearman rho), with the highest γδ-T cell levels observed in young patients. In flow cytometric analysis we found a strong Vδ2 dominance within the γδ-T cell population (*p* = 0.005, Spearman rho) (Figure 1A). Interestingly, we found no statistically significant associations between the γδ-T cell percentages and the disease activity markers (i.e., the Harvey-Bradshaw Index, faecal calprotectin, or C-reactive protein). This suggests that CD-mediated inflammation was not responsible for the observed changes in the γδ-T cell populations (Figure 1B).

**Table 1:** Summarised baseline characteristics.

Variable	All	Infliximab	Adalimumab
*n*	46	20	26
Females, *n* (%)	22 (48%)	10 (50%)	12 (46%)
Age, median (range)	38 (19–67)	39 (19–61)	36 (20–67)
Smokers, *n* (%)	15 (33%)	5 (25%)	10 (39%)
Ileal disease, *n* (%)	28 (61%)	11 (55%)	17 (65%)
Colonic disease, *n* (%)	40 (87%)	17 (85%)	23 (89%)
Steroid, *n* (%)	5 (11%)	4 (20%)	1 (4%)
AZA/6MP, *n* (%)	15 (33%)	3 (15%)	12 (46%)
M, *n* (%)	3 (7%)	2 (10%)	1 (4%)
Previous infliximab, *n* (%)	19 (41%)	8 (40%)	11 (42%)
Previous adalimumab, *n* (%)	9 (20%)	1 (5%)	8 (31%)

### In Vivo Clonal Expansion of γδ-T cells Induced by Infliximab

**Figure 2:**
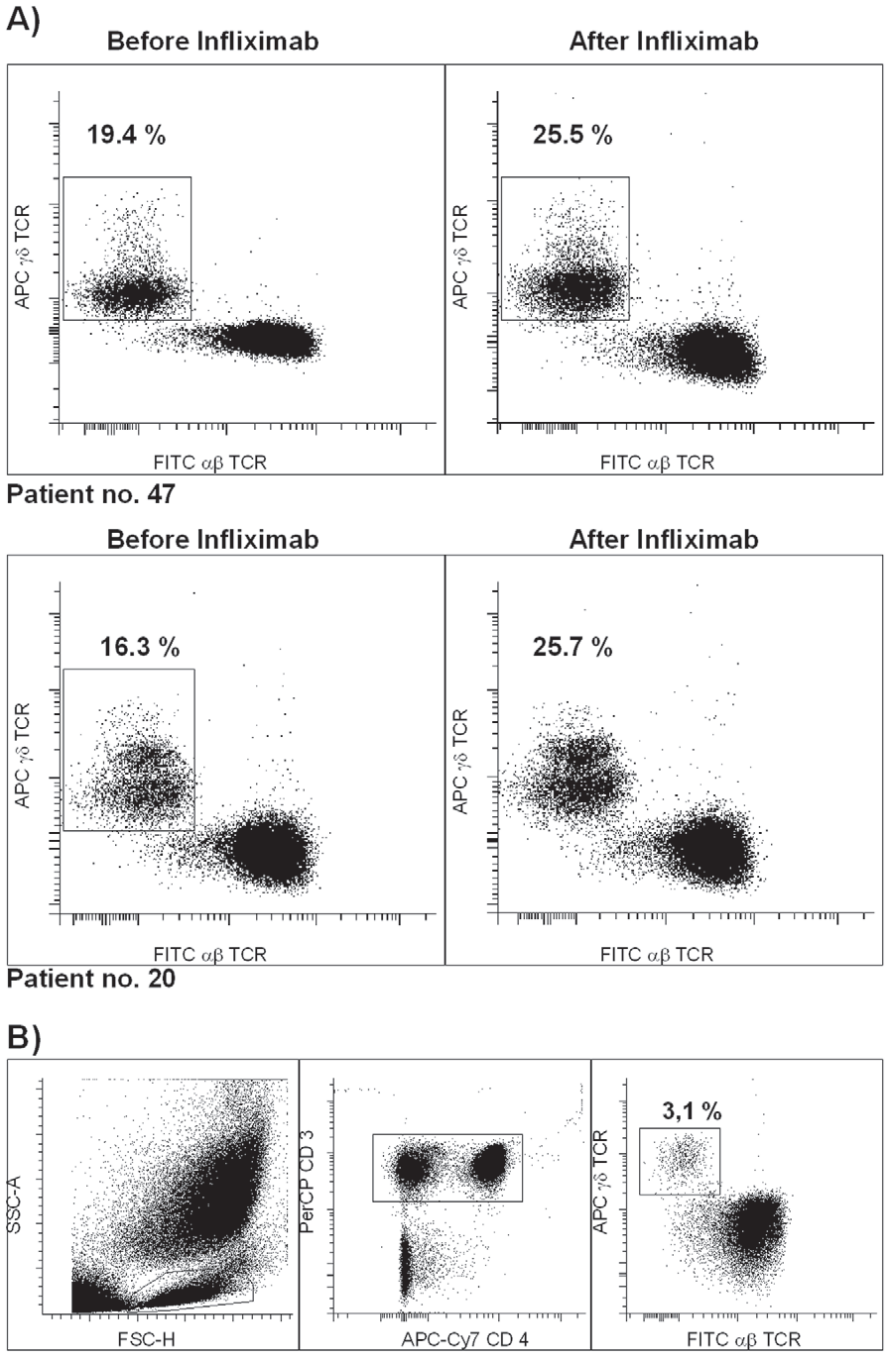
The γδ-T cell level of three CD patients who were treated with infliximab increased shortly after infliximab infusion. A) The results from two representative young male CD patients are shown. B) Illustration of the gating strategy. Using multicolour flow cytometry, γδ-T cells were identified by positive gating on live lymphocytes based on forward/side scatter (left panel), the selection of both CD4+ and CD4- cells among the CD3+ T cells (middle panel), and the separation of γδ-T cells from αβ-T cells (right panel).

Subsequently, we investigated the dynamic changes in γδ-T cell populations during anti-TNF-α therapy. To be able to identify the direct effects and separate them from the secondary immunological effects, we examined changes in γδ-T cells at early points during anti-TNF-α therapy. The majority of CD patients exhibited only minor fluctuations in γδ-T cell frequency. However, in the subgroup of young male patients with high baseline γδ-T cells, we observed a dramatic increase following treatment; in fact, the percentage of γδ-T cells increased significantly within the first 24 hours after a single infliximab infusion (Figure 2).

**Figure 3:**
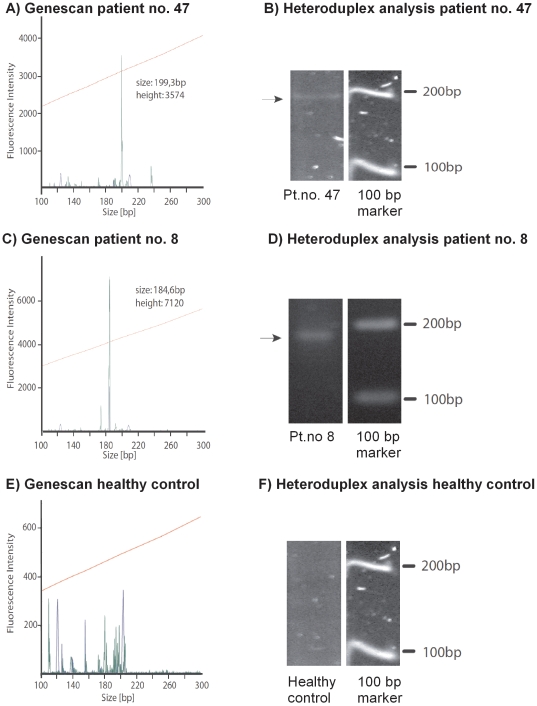
Analysis of *TCRD* rearrangement and heteroduplex analysis confirms the clonal expansion of γδ-T cells. For the fragment and heteroduplex analyses two representative examples of three CD patients are shown. A) Genescan analysis of patient number 47 shows a single monoclonal peak at 199 bp with a fluorescence intensity of 3574. B) Heteroduplex analysis of the same sample demonstrates a dominant band of approximately 200 bp. C) Genescan of patient number 8 shows a single monoclonal peak at 184 bp with a fluorescence intensity of 7120. D) Heteroduplex analysis of the same sample shows a very prominent band in the range of 180–190 bp. E) Genescan of a healthy control shows various polyclonal bands with different sizes and generally low fluorescence intensity. F) Heteroduplex analysis of the same sample resulted in a DNA smear with weak fluorescence intensity.

In an attempt to confirm our findings that young male patients with a high baseline percentage of γδ-T cells appear to be prone to further γδ-T cell expansion during treatment with immunosuppressive drugs, we examined additional patients outside the original CD cohort. We identified a 33-year-old male CD patient (number 47) who had been treated with infliximab monotherapy at 8-week intervals for 9 years. He presented with an extraordinarily high baseline frequency of γδ-T cells (18–21% of all CD3+ T cells). These results were confirmed by three measurements performed between infliximab treatments over a 6-month period of stable clinical remission. From this high baseline frequency, the γδ-T cell population expanded to 25% 2 days after infliximab infusion and to 36% 3 months after treatment. Clonotypically, 90% of the γδ-T cells were Vδ2, suggesting the existence of a predominant γδ-T cell clone. Genescan analysis, as well as heteroduplex analysis, confirmed the monoclonal expansion of a γδ-T cell clone in the peripheral blood of this patient (Figure 3 A, B).

While the expansion of γδ-T cells was observed in CD patients treated with infliximab, no significant changes were observed in adalimumab-treated individuals. This finding was surprising, as the majority of patients in this group had received adalimumab in combination with additional immunosuppressive agents, such as thiopurines or methotrexate. One male CD patient with γδ-T cell expansion during maintenance therapy with infliximab and azathioprine was later shifted to adalimumab. Interestingly, unlike patients on continued infliximab treatment, we detected no further γδ-T cell expansion in this ‘cross-over’ patient following the change in therapy to adalimumab.

### The Impact of Immunomodulators on γδ-T cells

In adalimumab-treated CD patients, the baseline level of γδ-T cells (mean 3.9%) was slightly higher than the baseline level in infliximab-treated CD patients (mean 2.7%) (*p* = 0.27, Student’s *t*-test). This finding may, in part, be explained by the slightly higher frequency of patients treated with immunomodulators in the adalimumab group. In CD patients that received azathioprine, 6-mercaptopurine, or methotrexate, the baseline γδ-T cell represented an average of 4.4% of all T cells, with values ranging from 2.1–6.7%; these results were not significantly different from those found in CD patients not treated with immunomodulators (*p* = 0.17, Student's *t*-test). However, these values were masked by a subpopulation of CD patients with unusually high levels of γδ-T cells. Repeated blood sampling from this subgroup revealed persistently high percentages of γδ-T cells and we observed 8.5% γδ-T cells in a patient (no. 8), receiving 6-mercaptopurine. Genescan analysis revealed a narrow oligoclonal γδ-T cell pattern with a dominant clone, which was confirmed by heteroduplex analysis (Figure 3 C, D). These results suggest that immunomodulators may induce proliferation and clonal selection of γδ-T cells.

### In Vitro Proliferation of γδ-T cells Induced by Anti-TNF-α Agents

**Figure 4:**
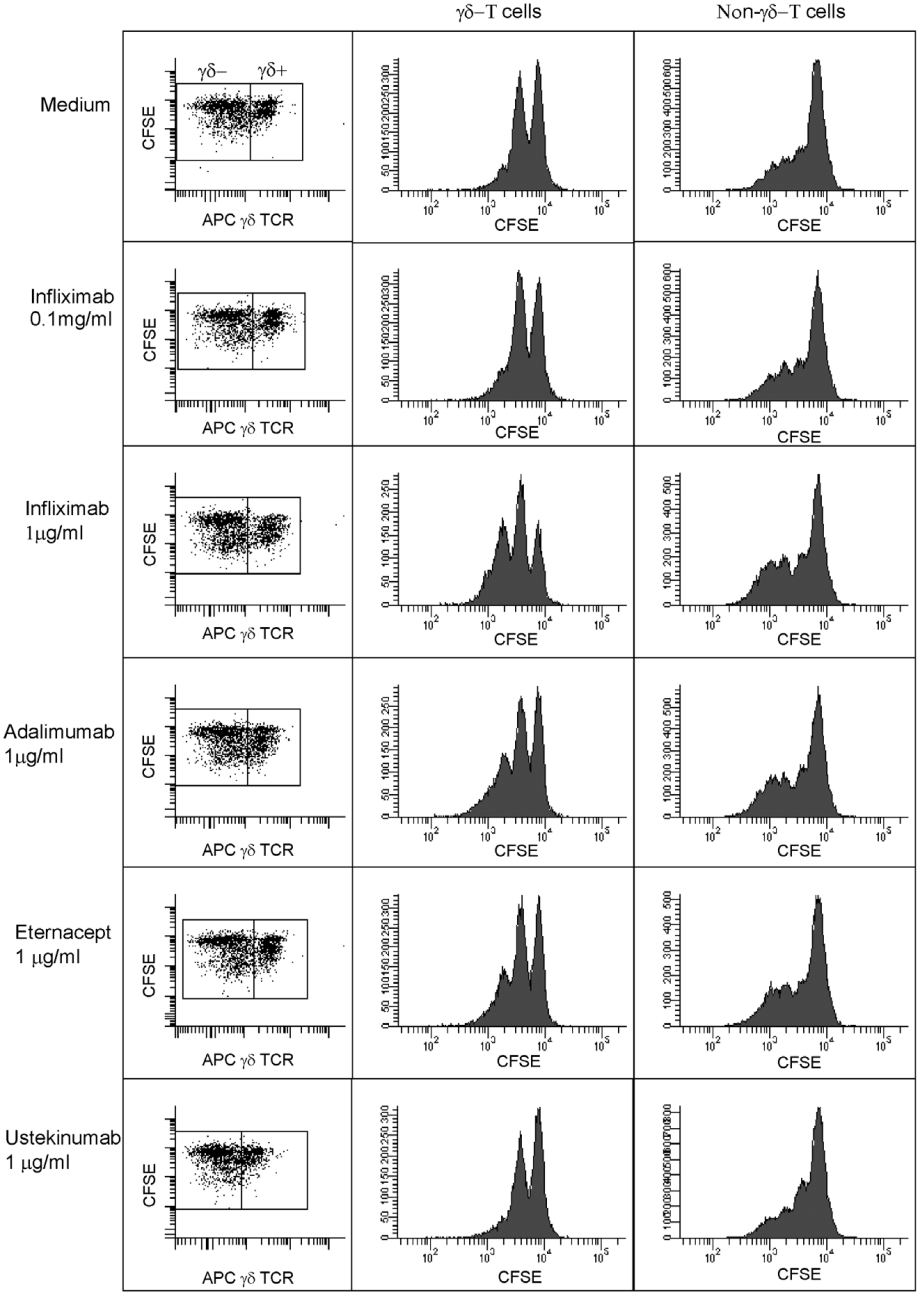
Anti-TNF-α agents promote the expansion of γδ-T cells in IL-2 stimulated PBMNC cultures. PBMCs were labelled with CFSE and were analysed by flow cytometry after 5 days of culture. γδ-T cells were stained with APC. The anti-TNF-α agents (infliximab, adalimumab, and etanercept) induced proliferation of γδ-T cells in vitro (middle panel), whereas the proliferation on non-γδ-T cells was negligible (right panel). In IL-2 -supplemented (200 IU/ml) PBMC cultures, infliximab (0.1 µg/ml or 1.0 µg/ml) induced a dose-dependent proliferation of γδ-T cells (middle panel). Etanercept induced proliferation of γδ-T cells in the same magnitude.The anti-IL-12p40 antibody ustekinumab had no effect on γδ-T cell proliferation in vitro. PBMCs from three CD patients and two healthy controls were analyzed.

In order to discriminate between the direct effects of anti-TNF-α agents on γδ-T cells and the secondary, indirect effects that are mediated through the modulation of the inflammatory cytokine milieu in vivo, we examined the impact of anti-TNF-α agents on γδ-T cells in vitro. An in vitro analysis of the effects of infliximab, adalimumab, and the TNF-α receptor fusion protein etanercept on γδ-T cells would also allows us to exclude the possibility that the observed increases in circulating γδ-T cells was caused by a redistribution of the intestinal pool of γδ-T cells. When PBMCs from CD patients and healthy controls were cultured for 5 days, the anti-TNF-α agents induced the dose-dependent, selective proliferation of γδ-T cells in cultures supplemented with IL-2 (200 IU/ml). This proliferative effect was reversed by the addition of recombinant TNF-α. Our findings with etanercept indicate that neutralisation of TNF-α in the culture medium, rather than the membrane-bound form of TNF-α, is involved (Figure 4). The induced proliferation was of the same magnitude for all anti-TNF-α agents. Proliferation was more pronounced in γδ-T cells compared to non-γδ-T cells, resulting in a relative increase in γδ-T cell frequency compared to other lymphocyte populations. Ustekinumab, an antibody against IL-12p40 that was used as a negative control did not affect the γδ-T cell frequency. Importantly, we observed a proliferative effect of anti-TNF-α agents on γδ-T cells from both CD patients and healthy controls, indicating that the proliferation of γδ-T cells in response to anti-TNF-α agents may not be restricted to CD patients.

## Discussion

The clinical rationale for combined immunotherapy in the management of CD is well established and has been strengthened by the results of the recent SONIC study [22], [23]. To date, the consensus is that the clinical benefits of immunomodulators outweigh the risk of lymphoma. Despite several case reports detailing the development of HSTCL in CD patients, it has not been possible to determine if the anti-TNF-α agents played a primary role in the lymphomagenesis or if HSTCL should be regarded as the result of a clonal evolution within the more generalised chronic inflammatory processes that characterise IBD.

In the present study, we hypothesised that clonal γδ-T cell evolution, presumed to precede neoplastic transformation into HSTCL, may be promoted by the repeated administration of anti-TNF-α agents. In approximately 75% of CD patients, we observed a γδ-T cell frequency within the normal range (1–3% of all T cells). However, in the remaining 25% of CD patients receiving immunomodulatory therapy, we observed a persistently high percentage of γδ-T cells; amongst these, young male patients appeared to be more prone to a clonal γδ-T cell expansion following repeated infliximab infusion. In addition, the analysis of *TCRD* rearrangements revealed a dominant clone in a female patient receiving monotherapy with 6-mercaptopurine, supporting the idea of stepwise clonal selection of γδ-T cells during combined immunotherapy.

These findings are difficult to interpret as being completely drug-induced as CD patients may have an increased number of oligoclonal γδ-T cells caused by the chronic inflammation associated with this disease [2], [3], [24]. One explanation could be γδ-T cell recruitment from the inflamed intestinal mucosa, where γδ-T cells are abundant. However, new evidence challenges this hypothesis due to the lack of identical γδ-T cell clones in the intestinal mucosa and peripheral blood of CD patients [24]. Furthermore, a direct neoplastic effect on γδ-T cells cannot be ruled out. In fact, whether the chronic inflammation itself is responsible for the development of lymphoproliferative disorders in CD patients is still a matter of contention within the field [12]. Hence, the increased number of γδ-T cells in CD patients may simply reflect the degree of intestinal inflammation, and patients with refractory or uncontrolled inflammation may therefore be overrepresented in the group receiving thiopurines. However, in our study we did not find a significant correlation between the percentage of γδ-T cells and key inflammatory markers. Furthermore, inflammation certainly cannot explain the massive increase in γδ-T cells seen after infliximab treatment in cases where the disease activity index and systemic and intestinal inflammatory markers declined.

This study was focused on investigating the impact of immuno suppressive therapy on γδ-T cells and its possible role in lymphomagenesis thus allowing the identification of at risk patients. Our results indicate that γδ-T cells can be modulated by anti-TNF-α therapy in vitro, which may represent a first step to substantiate a causal role of infliximab in γδ-T cell malignancies. In this context, we measured changes in the frequency of γδ-T cells in the peripheral blood of CD patients as early as 24 hours after infliximab infusion. Three male patients showed a marked response, nearly doubling their number of γδ-T cells. The observed changes in γδ-T cells appeared robust compared with the minor changes we observed in other lymphocyte subpopulations [25].In the light of the well-known preponderance of cases of HSTCL in males, it is noteworthy that our study only demonstrated high levels of γδ-T cell expansion in male CD patients. As a proof of concept, we analysed the peripheral blood of a CD patient who had undergone long-term treatment with infliximab (9 years). Interestingly, we found unusually high levels of γδ-T cells (on average 20% of all T cells), which increased further to 36% during the study period. Genescan analysis of *TCRD* rearrangements in cells from this patient showed a clonal expansion of γδ-T cells, which was confirmed by heteroduplex analysis. This finding, together with our in vitro data, suggests the infliximab-induced clonal expansion of γδ-T cells in the peripheral blood rather than a redistribution of the intestinal γδ-T cells. Furthermore, the expanded γδ-T cells were mainly of the Vδ2 subtype, and not δ-1, as one would expect if they were of mucosal origin [1]. However, we cannot yet say that this dominant clone actually represents a premalignant transformation as oligoclonal γδ-T cell populations are occasionally found in CD patients without the development of lymphomas [24]. Additionally, we do not have data confirming that the same γδ-T cell clone actually expanded as a direct consequence of repeated infliximab infusions. However, it is remarkable that the strongest increase in the proportion of γδ-T cells was seen in young males receiving infliximab.

The mechanisms underlying the action of thiopurines in the treatment of IBD are poorly understood [26]. The observed increased frequency of γδ-T cells following treatment with thiopurines may be a double-edged sword, as γδ-T cell expansion could represent a principal therapeutic mechanism in CD, while at the same time predisposing the patient to lymphoma. Gene expression profiling has recently established that γδ-T cell lymphomas have distinct molecular signatures [4]. In the future, various gene profiling strategies may be able to identify premalignant genetic lesions in CD patients receiving combined immunotherapy [27], [28].

The proliferation of γδ-T cells in vivo was not seen in patients treated with adalimumab. We believe that this could be the result of the chosen observation window, dosage, or method of administration (subcutaneous *vs.* intravenous) rather than a genuine difference in intrinsic therapeutic mechanisms. Our in vitro data suggest that the γδ-T cell expansion is a dose-dependent effect of multiple anti-TNF-α reagents. However, a valid in vivo comparison of the drugs is difficult to undertake, as the conventional clinical algorithm dictates that most CD patients on adalimumab will have received infliximab earlier. It should be noted, however that lower doses of infliximab are recommended (3 vs. 5 mg/kg) in the treatment of rheumatoid arthritis, which might explain the single case of HSTCL that has been reported in patients with rheumatoid arthritis.

We present an example of how epidemiological data can be translated to the patient level and further to the in vitro level. Our results confirm earlier findings that the number of γδ-T cells is increased in a subgroup of CD patients; however, in our study, the increased γδ-T cell frequency was independent of disease activity. A subset of CD patients treated with thiopurines or methotrexate had an extremely high baseline frequency of γδ-T cells. These patients appear to have a lower threshold for expansion of γδ-T cells when treated with infliximab, which we found to be a potent inducer of γδ-T cells in vitro. Further studies are warranted to substantiate our observation that inflixmab may promote lymphomagenesis by the repeated clonal expansion of γδ-T cells.
